# Assessment of Risk Factors in Synanthropic and Wild Rodents Infected by Pathogenic *Leptospira* spp. Captured in Southern Chile

**DOI:** 10.3390/ani10112133

**Published:** 2020-11-17

**Authors:** Jhuliana Luna, Miguel Salgado, Carlos Tejeda, Manuel Moroni, Gustavo Monti

**Affiliations:** 1 Graduate School, Faculty of Veterinary Sciences, Universidad Austral de Chile, Valdivia 5090000, Chile; jhuly_kath@hotmail.com; 2 Preventive Veterinary Medicine Institute, Faculty of Veterinary Sciences, Universidad Austral de Chile, Valdivia 5090000, Chile; miguelsalgado@uach.cl (M.S.); carlostb81@gmail.com (C.T.); 3 Veterinary Pathology Institute, Faculty of Veterinary Sciences, Universidad Austral de Chile, Valdivia 5090000, Chile; manuelmoroni@uach.cl

**Keywords:** leptospirosis, rodents, infection status

## Abstract

**Simple Summary:**

Leptospirosis is a zoonosis caused by pathogenic *Leptospira*, and synanthropic and wildlife species of rodents are an important source of infection; however, much of the information about the progression of the infection was obtained from lab murine models. The aim of this study was to assess infection status and risk factors by pathogenic *Leptospira* in synanthropic and wild rodent species and describe histopathological lesions in several organs from naturally infected animals. In this study, 121 rodents from three synanthropic species and two wild species were trapped within dairy farms in Southern Chile, where the bacteria were present. Liver, heart, kidney, and lungs from trapped animals were analyzed by different techniques to detect if the lesions present were produced by the bacteria. A large proportion of animals were identified as infected that were not detected by the microscopic agglutination test. There is a lower risk of infection in the fall compared to the rest of the seasons, and the synanthropic species has a lower risk of infection in comparison with wildlife species. Immunohistochemistry and quantitative real-time lipL32 polymerase chain reaction contributed to identifying the presence of pathogenic *Leptospira* in related histological lesions and 50% more infections than serology.

**Abstract:**

Leptospirosis is caused by pathogenic *Leptospira*, and synanthropic and wildlife species of rodents are an important source of infection; however, much of the information about infection progression was obtained from murine models. The aim of this study was to assess infection status and risk factors associated with pathogenic *Leptospira* in synanthropic and wild rodent species and describe histopathological lesions in several organs from naturally infected animals. In a cross-sectional study, 121 rodents from three synanthropic species and two wild species were trapped in dairy farms in Southern Chile. Liver, heart, kidney, and lungs from trapped animals were fixed in formalin and stained with hematoxylin–eosin. Tissues with lesions consistent with *Leptospira* infection were tested by immunohistochemistry (IHC) and real-time polymerase chain reaction (qPCR) using the *LipL32* antigen. Risk factors were assessed by a conditional mixed-logistic regression model. More than half (56.7%) of the negative reactors to the microscopic agglutination test were identified as infected either by IHC/qPCR. A lower risk of infection compared to the rest of the seasons was found in the fall, and the synanthropic species have a lower risk of infection in comparison with the wildlife species. IHC and qPCR contributed to the identification of pathogenic *Leptospira* in related histological lesions and 50% more infections than serology.

## 1. Introduction

Leptospirosis is one of the most widespread zoonotic diseases caused by infection with pathogenic spirochetes of the *Leptospira* genus [1]. Human patients usually present with a nonspecific self-limiting febrile illness; however, a variable proportion of cases develop a severe disease called Weil’s disease, which is characterized by jaundice, acute renal failure, hemorrhagic diathesis, and severe pulmonary hemorrhagic syndrome (SPHS) [2]. The main burden of leptospirosis is caused by severe disease forms and can vary among countries. Nonetheless, in countries in which leptospirosis is endemic, case fatality from Weil’s disease is >10% and >50% for SPHS [2,3].

In humans, transmission could directly or indirectly come into contact with contaminated urine of infected animals, and several domestic and wild animals are maintenance or incidental reservoirs. Synanthropic rodents (*Rattus norvegicus* (RN), *R. rattus* (RR), and *Mus musculus* (MM)) are considered important sources of infection by pathogenic *Leptospira*, among which RN is the main host of *L. interrogans* serovar Copenhageni, which is one of the most pathogenic *Leptospira* species for human infections [4]. Other important sources of infection for humans are domestic dogs, cattle, horses, sheep, and goats [1]. Rodents are persistent renal carriers but rarely develop signs of the infection [5].

Much of the information about the progression of the infection has been obtained from murine models [6,7]. Paradoxically, it has been difficult to reproduce the disease in rodents that are naturally infected; therefore, it has been difficult to associate lesions in experimentally infected rodents with findings in wild rodents [8].

The primary lesion in leptospirosis-affected animals and humans damages the membranes of the blood vessel endothelial cells [9]. Systemic vasculitis is one of the main mechanisms of tissue damage resulting from direct pathogen invasion, immune complex deposition, autoantibodies, and cell-mediated immunity; however, it is not always reported in leptospirosis [10]. In naturally infected rodents, lesions, such as tubulointerstitial nephritis and the infiltration of lymphocytes, macrophages, and plasma cells surrounding small cortical arteries in the renal cortex [8,11,12], have been reported. In naturally infected animals, including experimental rodents or humans, liver, lung, and heart lesions [10,13,14,15,16] have been described.

Given the importance of rodents as a source of infection of pathogenic *Leptospira* to humans, and due to the lack of pathological information in naturally infected rodents, the aim of the present study was to assess infection status and risk factors associated with pathogenic *Leptospira* in five synanthropic and wild rodent species and to describe histopathological lesions in several organs of the infected animals.

## 2. Materials and Methods

### 2.1. Study Areas and Design

**Figure 1:**
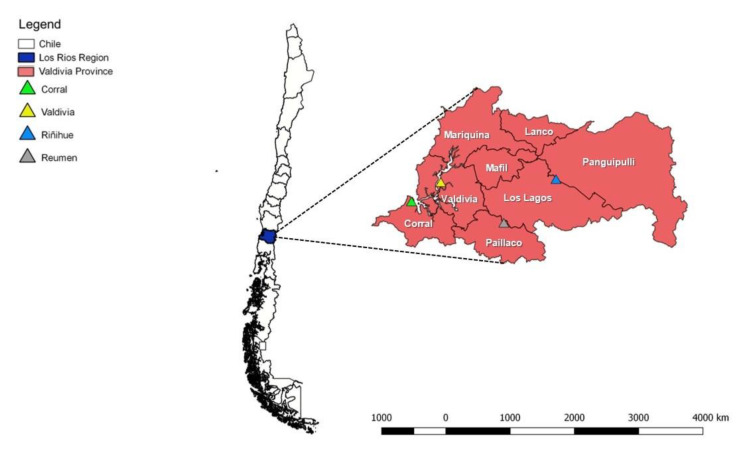
Dairy farms on which rodent sampling was performed, Region de Los Ríos, Valdivia, Chile.

A cross-sectional study in which 121 rodent species were trapped on 11 dairy farms in the Los Ríos Region, Southern Chile, was designed. Most of these farms did not apply any systematic control with rodenticides. Capturing was conducted within Valdivia Province, Southern Chile, and included the rural communities around the city of Valdivia (39° 47′ 21.48″ S 73° 14′ 37.32″ W), Reumen (39° 59′ 54.96″ S 72° 49′ 18.12″ W), and Riñihue (39° 46′ 25.32″ S 72° 28′ 20.28″ W) (Figure 1).

Three synanthropic species (MM, black rat (RR), and brown rat (RN)) and two wild species *Abrothrix olivaceus* and *A. longipilis* (AO and AL, respectively) were targeted. The general topography in this area consists of rolling hills with open pastures, woods and bushes, and small ponds and streams. Areas included in this study consisted of a combination of these features and various types of buildings (barns, sheds, and outbuildings).

### 2.2. Rodent Trapping

Between August 2014 and December 2015, one hundred traps (20 cm × 20 cm × 60 cm Tomahawk cages) were located once in the period on farms in the areas on which cattle feed and dwell and rodents were usually seen in addition to setting the traps along wild forests paths based on indicators of rodent activity. Traps were positioned at distances of 50 to 150 m with at least 5 m spacing between cages. Traps were baited with oatmeal and vanilla flavoring, set in the afternoon, and examined for four days. They were checked daily in the morning. Endangered, threatened, or protected species were immediately released.

Trapped animals were transported to the Veterinary Pathology Institute, Faculty of Veterinary Sciences, Universidad Austral de Chile, in which they were euthanized and processed. Capture, management, and euthanasia of rodents were conducted in compliance with the specifications of the American Society of Mammologists. The authors confirm that the ethical policies of the journal, as noted on the journal’s author guidelines page, were followed, and the appropriate ethical review by the Animal Care Ethics Committee of the Universidad Austral de Chile (committee approval #53/2013). Nontarget, native animals were released immediately after identification.

### 2.3. Identification and Sample Collection

Once arriving at the necropsy facility, animals were anesthetized with isoflurane and euthanized by an overdose of ketamine–xylazine, which was administered intraperitoneally (IP) [17]. Euthanized rodents were then described by their morphometric characteristics, namely sex, maturity, weight, and various lengths, including head–body, tail, and ear pinnae. The skin was examined for the presence of ticks, fleas, and lice. Biological samples were obtained immediately after euthanasia. To avoid cross-contamination, several measures were taken, in which an individual disposable sterile scalpel was used for each animal that was sterilized with a Bunsen burner during the necropsy to take samples from different organs of the same animal.

Based on phenotypic characteristics (ears, body and tail length, fur color, and sex), each animal was identified by genus, species, and gender. In addition, age was estimated using body length and weight, and they were classified as juvenile or adult [18].

Blood samples were collected by cardiac puncture (up to 1 mL) with individual disposable sterile syringes and transferred to vacutainers. One kidney from each animal was aseptically removed for polymerase chain reaction (PCR) testing. Liver, spleen, the remaining kidney, and lungs were removed aseptically and fixed in 10% neutral-buffered formalin at least for 72 h, and tissues were then stained by hematoxylin–eosin (H&E). Blood was centrifuged, and serum and harvested tissues were stored at −20 °C for later PCR experiments.

All personnel responsible for trapping, handling, and performing necropsies used a biosecurity protocol and norms established by the funding agency (Manual of Biosecurity Norms FONDECYT-CONICYT; CONICYT 2008) using appropriate equipment. Carcasses were disposed of according to the dangerous biological protocols of the University (manual for managing biological waste at Universidad Austral de Chile).

### 2.4. Diagnostic Tests and Infection Status

#### 2.4.1. Serological Testing

A microscopic agglutination test (MAT) was performed as previously described using a reference panel of six pathogenic serovars: (1) *L. interrogans* serovar Pomona, (2) serovar Canicola, (3) serovar Icterohaemorrhagiae, (4) serovar Autumnalis, and (5) *L. borgpetersenii* serovar Ballum, and (6) serovar Hardjo [19]. Serum samples were considered positive at a reciprocal titer of ≥1/100. This test was run at the Infectious Diseases Laboratory, Preventive Veterinary Medicine Institute, Faculty of Veterinary Sciences, Universidad Austral de Chile.

#### 2.4.2. Histological Processing

After paraffin inclusion, 4 mm paraffin sections were cut and stained with H&E following the procedure reported by [20] and examined using a conventional microscope with 10×, 40×, and 100× lenses in the laboratory of the Veterinary Pathology Institute, Faculty of Veterinary Sciences, Universidad Austral de Chile. Cross-contamination was avoided by cleaning the microtome leaves with xylene between cuts of each sample. The lesions were classified first as consistent with pathogenic *Leptospira* infection or not [8,9,13] and then interstitial nephritis was classified according to severity levels [20]:

1. Mild: when interstitial nephritis with inflammatory infiltrate rich in macrophages and lymphocytes restricted to perivascular areas without expansion to other areas of kidney is observed.
2. Moderate: if interstitial nephritis that consisted of inflammatory infiltrate with the predominance of lymphoplasmocitary cells are present with 1 to 2 lesions per field (magnification 40×). In addition, isolated inflammatory spots are present.
3. Severe: if interstitial nephritis that consists of inflammatory infiltrate with predominance of lymphoplasmocitary cells are present with more than 1 to 2 lesions per field (magnification 40×). In addition, inflammatory spots may be present.

#### 2.4.3. Detection of Pathogenic Leptospira Antigens in Tissue Samples

Given that histological findings for leptospirosis are nonspecific, the diagnostic strategy was targeted to the detection of leptospiral antigens in several tissues. Tissues with lesions consistent with *Leptospira* infection were selected for being tested by immunohistochemistry and qPCR in order to confirm pathogenic *Leptospira* infection in the observed lesion.

#### 2.4.4. Immunohistochemistry (IHC)

Serial 4 μm sections of kidney were placed on positively charged slides, and antigen retrieval was performed with a microwave oven at 600 W. Samples were incubated in a blocking solution of 2% skimmed milk for 20 min. A polyclonal rabbit antiserum specific for the leptospiral major outer membrane protein LipL32 [7] was used to detect the leptospiral antigen. The sections were incubated overnight with a 1:1000 dilution at room temperature. Antibody binding was detected by a horseradish peroxidase-labeled streptavidin–biotin polymer kit (Universal LSAB2 Kit-HRP; Dako (Dako LSAB+Kit, peroxidase streptavidin), Santa Clara, CA, USA), and diaminobenzidine (DAB) as the chromogen. Finally, a hematoxylin and methenamine-silver-modified counterstain was applied. A positive reaction was identified by the brown-stained bacterium or *Leptospira* aggregations over the tissue [21] as observed with a conventional microscope (CX41F, Olympus, Tokyo, Japan) with the M Shot Image Analysis System software, v 1.0 (MSHOT, Guangzhou, China).

Kidney obtained from infected cattle with pathogenic *Leptospira* spp. was used as the positive control, and a serial section incubated with distilled water was used as a negative control.

#### 2.4.5. Molecular Identification of Pathogenic Leptospira spp.

Tissue samples were subject to a DNA extraction–purification protocol using the High Pure PCR Template Preparation kit (Roche, Indianapolis, IN, USA), following the manufacturer’s instructions.

The DNA templates obtained from the above protocol were analyzed in a qPCR system (Roche LightCycler 2.0) using a TaqMan probe targeting the *LipL32* gene [22], which is specific only for pathogenic *Leptospira* spp. The primers lipL32-45F (5′-AAG CAT TAC CGC TTG TGG TG-3′) and lipL32-286R (5′-GAA CTC CCA TTT CAG CGA TT-3′) were used to amplify a fragment of 242 bp, which was detected by the above-mentioned probe, LipL32-189P (FAM-5′-AA AGC CAG GAC AAG CGCCG-3′-BHQ1). The amplification mixture for each sample contained 0.7 μM primers, 0.15 μM probe, 10 μL Master Mix TaqMan universal (Roche), and 5 μL DNA template in a total volume of 20 μL. Samples were amplified with the following program: (1) initial denaturation at 95 °C for 2 min, (2) 40 cycles of denaturation for 5 s at 95 °C, and (3) annealing/elongation for 30 s at 58 °C. The system contained a negative and positive control in order to survey the proficiency of the reaction in addition to negative and positive DNA controls.

#### 2.4.6. Definition of Infection Status

Given that several tests were applied and to increase the overall sensitivity of the diagnostic algorithm, the final infection status was determined by a positive reaction to MAT and/or pathogenic *Leptospira* detection either by positive IHC or qPCR on any tissue.

### 2.5. Statistical Analysis

Association between histopathology findings and infection status was tested by chi-square and Fisher’s exact tests (*p* < 0.05), after which the 95% confidence interval was estimated.

The potential association between some animal characteristics and whether the animal was infected or not was assessed by a mixed random intercept-logistic regression model. The strategy for building the model consisted of the initial screening of individual variables, including species, gender, age, and season in which an animal was trapped, by obtaining unconditional models. The farm on which an animal was trapped was treated as a random effect to assess variability between locations, and the remaining variables were treated as fixed effects. Variables associated with the outcome (*p* < 0.25) were eligible for inclusion in the conditional model that was built using a forward variable selection method, including potential interactions. A comparison of the goodness-of-the-fit between different models was assessed using the Akaike Information Criteria (AIC) index. Finally, confounders were assessed based on their biological significance. Statistical significance was set at *p* < 0.05. Mixed-effects logistic regression was run using the lme4 package [23]. All analyses were performed using R (V3.2.1) [24].

## 3. Results

A total of 121 rodents from all target species were captured. Most (64.5%) were represented by synanthropic species, mainly RR and the rest (35.5%) wild, represented by AO. Considering overall captures, 69% of the rodents trapped were juveniles, and 60% were male, but no statistically significant differences between any groups were found (Table 1).

The number of rodents trapped per farm ranged from 5 to 24 (mean = 11; SD = 6.5) and the distribution of species captured differed significantly across the farms (*p* < 0.01). More than 20 were trapped in two farms, two of them between 10 and 20, and the rest between 5 and 10.

Enough blood was obtained for serological testing in 104 out of 121 captured rodents. From them, 13.5% (95% CI: 13.2–15.1) tested MAT-positive. Almost 29% (95% CI: 27.2–29.9) of the wild species tested MAT-positive, while 5.8% (95% CI: 3.7–7.9) of the synanthropic rodents that tested positive, excluding MM. The seroprevalence among different species was 30.0% (95% CI: 28.6–34.6) for AO, 20.0% (95% CI: 28.6–34.6) for AL, 5.1% (95% CI: 4.9–9.9) for RN, and 8.0% (95% CI: 7.4–15.2) for RR. The seroprevalence by gender was 5.0% (95% CI: 4.8–9.7) and 18.8% (95% CI: 18.3–21.2) for females and males, respectively, and by age with adults 15.2% (95% CI: 14.7–18.8) and juveniles 12.1% (95% CI: 11.7–15.0). No significant differences based on age and gender regarding MAT results were found.

In general, the most frequent serovars detected were *L. borgpetersenii* serovar Hardjo (7.1%), *L. interrogans* serovar Pomona (64.3%), and Autumnalis (28.6%), with reciprocal titers ranging from 1/100 to 1/800. For AO, all animals reacted to *L. interrogans* serovar Pomona with reciprocal titers ranging from 1/100 to 1/800, AL reacted to *L. borgpetersenii* serovar Hardjo with a reciprocal titer of 1/400, RR reacted to *L. borgpetersenii* serovar Hardjo, *L. interrogans* serovars Pomona, and *L. interrogans* serovar Autumnalis with reciprocal titers ranging from 1/200 to 1/400, and RR reacted to *L. interrogans* serovar Autumnalis with titers ranging from 1/100 to 1/200.

In 98.4% of the trapped rodents (119/121), at least one lesion consistent with pathogenic *Leptospira* spp. infection based on H&E histopathology was observed. Table 2 indicates the distribution of the different types of lesions detected.

Out of the 476 slides processed (119 individuals with four slides for each one), a total of 323 presented any type of lesion compatible with pathogenic *Leptospira* spp. infection. The distribution of positive slides based on tissue was 106 from kidneys (32.8%), 105 from lungs (32.5%), 78 from liver (24.1%), and 34 from heart (10.55%).

**Figure 2:**
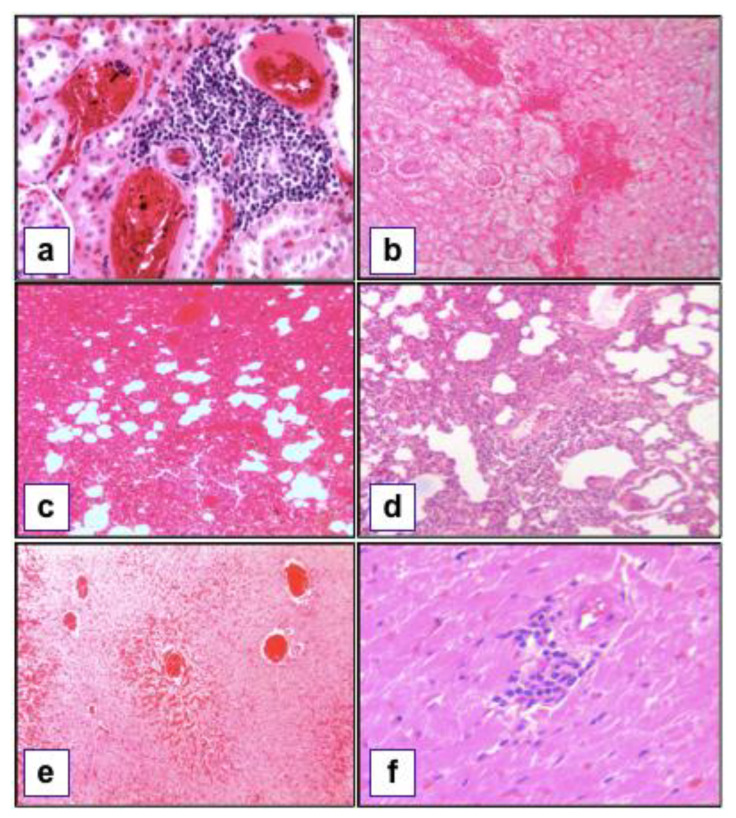
Histopathological findings compatible with pathogenic *Leptospira* spp. infection. (**a**) Lymphoplasmacytic, tubulointerstitial nephritis (H&E, 10×); (**b**) multifocal to coalescing areas of hemorrhage in the renal cortex (H&E, 10×); (**c**) diffuse hemorrhage in the lung parenchyma (H&E, 10×); (**d**) diffuse, lymphoplasmacytic alveolitis (H&E, 10×); (**e**) sinusoidal dilatation and congestion in the liver (H&E, 10×) (**f**) mild, lymphoplasmacytic myocarditis (H&E, 40×).

Most (87.6%) of the kidney slides presented a lesion; the most frequent lesions were lymphoplasmacytic infiltration of varying severity and hemorrhagic areas in the renal cortex (Figure 2a,b).

In 86.8% of the lung samples, varying degrees and extensions of hemorrhaging, congestion, and inflammatory mononuclear infiltration were noted. Additionally, parasite-associated granulomas with central necrosis, eosinophilic inflammatory infiltrate, hemosiderin, and hypertrophy of arterial walls were also observed (Figure 2c,d).

In addition, 64.5% of the liver slides were classified as consistent with pathogenic *Leptospira* spp. infection due to the presence of mononuclear infiltrates around blood vessels and bile ducts and circulatory changes, including sinusoidal congestion and hemorrhages (Figure 2e).

Lastly, 26.1% of the heart samples showed lesions consistent with the infection, mainly in the form of multifocal, mononuclear inflammation and hemorrhages in the myocardium (Figure 2f).

### 3.1. Identification of Pathogenic Leptospira by Immunohistochemistry (IHC) and Quantitative Real-Time lipL32 Polymerase Chain Reaction (qPCR)

From the 119 rodents with H&E histopathology findings, 31.9% (*n* = 38) reacted positively to the bacterium in at least one of the organs based on IHC. In contrast, 49.6% (*n* = 59) of the animals tested positive for qPCR in at least one of the organs and when any positive results of either method are interpreted as infected 63.9% (*n* = 76) of the rodents that tested positive (Table 3).

**Figure 3:**
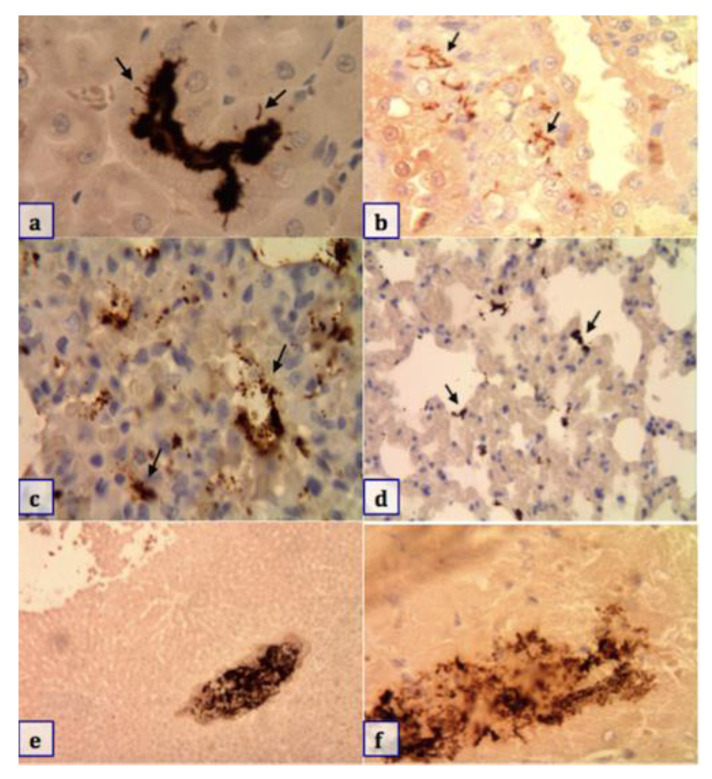
Indirect immunostaining (Anti-LipL32) of pathogenic *Leptospira* spp. Brown-stained aggregates of filamentous bacteria consistent with *Leptospira* (pointed out with arrows) in different tissues. (**a**,**b**) Tubular lumen and between cells of the tubular epithelium in the kidney (100×); (**c**,**d**) lung tissue (10×); (**e**) liver tissue (10×); (**f**) heart tissue (100×).

Based on IHC, the presence of pathogenic *Leptospira* was detected in 21.7% of the kidneys samples, mainly localized in the lumen of proximal convoluted tubules in the renal cortex; 16.2% of the lung samples had antigenic aggregates in the form of small cocci that were widely scattered in the parenchyma, and less frequently, filamentous aggregates were identified in samples of liver (9%) and heart (5.9%), as shown in Table 4 and Figure 3.

qPCR positive samples were more frequent in kidneys (45.3%) followed by lungs (14.3%) and less frequent in heart and liver (5.9% and 5.1%, respectively), as shown in Table 4. Moreover, 16.8% (20/119) of the samples tested positive in both tests (qPCR and IHC), and 12 samples tested positive according to qPCR and IHC in the same organ and lesion (mainly in kidneys but also in lung and liver lesions).

### 3.2. Infection Status of Trapped Rodents

Ninety-three percent of MAT-positive reactors (13 out of 14)) were also identified as infected either by IHC or qPCR; however, 56.7% (51/90) of the negative reactors to MAT were also identified as infected either by IHC or qPCR.

According to our definition of infection, 63.6% (77 out of 121) of rodents were infected. The species more frequently infected were AL (24%) and RN (17.4%), as shown in Table 5.

In general, H&E histopathological lesions consistent with pathogenic *Leptospira* spp. infections were present in a great proportion of infected rodents but were also frequently found in noninfected animals. For example, in 74% of the kidney slides from infected rodents, it was possible to identify interstitial nephritis lymphoplasmocitary; however, it was also found in noninfected animals, albeit less frequently (50%). More detailed information is shown in the Supplementary Files (Table S1).

The main pathological changes observed in affected animals included mononuclear inflammatory infiltrates in kidneys, liver, and heart, and hemorrhages in the lung. These lesions were found predominantly in infected male rodents of the AO species (Table 6, Table 7 and Table 8). These lesions were more frequent in infected juveniles except for lung hemorrhage, which was predominant in adults. However, only interstitial nephritis of different severity was statistically significant between either species, sex, or age groups (Table 6, Table 7 and Table 8).

Although more severe and moderate interstitial nephritis was observed in MAT-positive rodents reacting to serovar Pomona, rodent reactions to serovar Autumnalis did not show interstitial nephritis, but this difference was not statistically significant (*p* > 0.05).

In rodents with MAT reciprocal titers of 1/100, no inflammatory renal lesion was found. In contrast, in samples with reciprocal titers larger than 1/400, mild and moderate (28.6% and 57%, respectively) nephritis was frequently observed. However, only severe tubulointerstitial nephritis was statistically associated with reciprocal titers of 1/200 (*p* < 0.05).

### 3.3. Risk Factors Associated with Pathogenic Leptospira spp. Infection

For unconditional models, species (*p* < 0.001), season (*p* = 0.12), and sex were the variables primarily associated with infected status and used for building the conditional model. Results from the conditional mixed-logistic regression model are summarized in Table 9, and this model included two variables: (1) species (collapsed in synanthropic (MM, RR, and RN)) versus wildlife (AO and AL), season, and (2) farm on which rodents were trapped as random effects. The results suggest that during the fall, a lower risk of infection in comparison with the rest of the year was observed, and the synanthropic species had a lower risk of infection in comparison with wildlife species, which was an unexpected but interesting finding. No interactions or confounding effects were found to be statistically significant. The variance of the random effect was statistically not significant.

## 4. Discussion

Rodents represent the most abundant and diverse order of living mammals, accounting for approximately 40% of the total number of mammal species [25]. Due to the close relationship that these species have with human populations and domestic animals, they are considered important vectors in the transmission of leptospirosis [26].

The proportion of seroreactive animals found in this study was relatively low (13.5%), in particular in comparison with previous findings from Latin American countries, such as Brazil (68%) [27] or Colombia (82.7%) [28]. This finding probably resulted from the environmental and climate conditions of those tropical countries and their contribution to maintaining the pathogen for longer times in the environment during the seasons. However, 64% of the trapped infected rodents during the study confirmed the presence of pathogenic *Leptospira* spp. in rodent populations in the area, and this value was higher than those found in previous studies in the same region (20.4% and 37.8%) [29,30] and in other areas of the country [31]. Nevertheless, a comparison should be made with care given that, in the present study, other tissues were included for testing, such as lung, liver, and heart, which allowed us to increase the number of infected rodents. In addition, 49 of the positive animals based on kidney PCR did not present seroreactivity in MAT, which reinforced the value of qPCR for detecting carriers, which often have low titers of antibodies.

The Los Ríos Region is an area characterized by heavy and constant rain throughout the seasons with a yearly mean rainfall of 2588 mm and a yearly mean relative atmospheric humidity of 82%, which provides favorable conditions for pathogenic *Leptospira* spp. persistence in the environment. The season during which the animals were trapped was found as a factor associated with the risk of infection. In our study, rodents trapped in the fall had a lower risk of being infected in comparison with those trapped in the rest of the year. A seasonal effect was reported in the same area [29], but it was found that rodents trapped in the spring had higher odds of being infected in comparison to the summer. These differences might be explained by the variations in infected species among studies as the predominant species in the previous study was a synanthropic one (RR). In our study, a wildlife species (AO) was predominant, which had continuous minimum activity during the winter and maximum during the summer, while during the spring, the population structure is larger [32]. Although the largest proportion of captures in the study were in the spring, the distribution of the captures in the rest of the seasons was quite balanced; therefore, this balance did not affect the robustness of the results given that differences are highly significant. However, it was not expected that a wildlife species would show a higher risk of infection than synanthropic species in contrast to other reports from Chile [31], which showed that RN was the species with the higher proportion of renal carriers.

Worldwide, rodent infections are frequently associated with serovars Autumnalis, Ballum, Bim, and Arborea, and in rats, infections are associated with Copenhageni and Icterohaemorrhagiae [5]. However, in this study, we identified animals reacting to serovars, such as Pomona and Hardjo, which were previously reported in Chile as important causes of infection in the Los Ríos Region [33]. These findings might suggest the close relationship that rodents maintain with other domestic animals, such as cattle or other domestic animals, which usually live on area farms, was also reported in another study [34].

This study reports the infection status and describes histological lesions in synanthropic and wild species of rodents naturally infected with pathogenic *Leptospira*, which were captured in dairy farms in the Los Ríos Region in Southern Chile. A wide range of morphological alterations was detected in some of the organs of rodents naturally infected with pathogenic *Leptospira* that were captured in dairy farms in the Los Ríos Region, Chile.

The lesions observed in the kidneys of these rodents have already been reported for pathogenic *Leptospira* infection [14,35]. The most common detected lesion was lymphoplasmacytic inflammatory infiltrate, which has been reported by others [12,35,36]. The inflammatory infiltrate is a primary lesion during acute renal injury in leptospirosis and can be caused by direct damage to the host tissue by *Leptospira* or the presence of the Leptospiral antigen, which initiates a renal immune response [37].

Previous studies evaluating renal morphology from urban rats carrying pathogenic *Leptospira* spp. have all reported that interstitial nephritis was the principal change attributed to pathogenic *Leptospira* spp. [8,36,38]. The pathogenesis of naturally infected rodents has not been extensively studied and is complicated by a wide array of potentially confusing environmental factors that may also induce pathology in wild rodents [12,35]. The histopathological lesions present in the kidneys of the rodents plus the positive results of the qPCR and IHC confirm that these rodents are carriers of pathogenic *Leptospira* and there is a high chance that they are a source of infection for people and other domestic animals on the farms. In the study area, favorable environmental conditions, such as contaminated soil or water, aided the survival of leptospires for prolonged periods [31,39,40].

The disease process in general is the same for all species or the infecting serovar of pathogenic *Leptospira* [9]. Although rodents are susceptible to infection, it is generally accepted that they are asymptomatic carriers of pathogenic *Leptospira* spp. [36]. As in the present study, tubulointerstitial nephritis is the most frequently found lesion in renal tissues from naturally infected rodents [11,12,35]. However, this type of nephritis was found also in about 50% of the animals that were not identified pathogenic *Leptospira*, and this finding could indicate sequelae of chronic infections in which pathogenic *Leptospira* spp. has already disappeared from renal tissue, but the lesion is still present. Another interesting finding was that 90% of the rodents that presented tubulointerstitial nephritis did not react serologically to MAT, which could be explained by the chronic phase of the infection. This last explanation could explain the statistical association between the presence of severe tubulointerstitial nephritis and relatively low serological reciprocal titers to MAT (1/200) found in this study [41]. In contrast, positive seroreactor rodents, in which the pathogen was identified in organs other than the kidney, also presented reciprocal titers up to 1/800 to MAT and were in a more acute phase of the infection [41].

High mortality rates can be partially attributed to the presentation of the SPHS that in several places replaces Weil’s syndrome as the main cause of death from leptospirosis [3,42], and primary lung lesions caused by leptospirosis have been described in early phases of the infection process [9,43]. However, lung lesions as found in the present study were not reported in naturally infected rodents before, but the most frequently lung lesions found in this study, such as pulmonary hemorrhage (68.8%) and mononuclear infiltrates (49.4%), were also reported in experimentally infected mice [13,43]. Conversely, in the present study, pulmonary hemorrhage was also found in noninfected rodents (61.4%), suggesting that causes other than infection by pathogenic *Leptospira* spp. could be associated with, for example, rodenticide poisoning. In addition, minimal information about heart lesions in naturally infected rodents exists. In our study, interstitial myocarditis was a relatively frequent finding that indicates histological evidence for human death by acute leptospirosis, however, it remains unclear if pathogenic *Leptospira* spp. has a predilection for cardiac tissue [44].

Although it was not statistically significant, infected AO rodents presented a tendency to present more inflammatory lesions in the kidney, liver, and heart, in addition to pulmonary hemorrhages than in the other species. Scarce information about the susceptibility/resistance to developing histological lesions by gender exists, but it has been reported that naturally infected animals of certain species and age are more susceptible to develop histological changes [9]; however, differences in developing lesions in laboratory strains of rodents were found [7]. In relation to age in our study, juvenile rodents presented a larger proportion of lesions than adult animals, and it has been suggested that the main pathological alterations occurred after the loss of passive maternal immunity [9]. Moreover, in murine models of different ages, the animals exhibited inflammatory liver lesions characterized by mononuclear infiltrate only in animals younger than four weeks old [13]; meanwhile, in animals older than 10 weeks, the dissemination of pathogenic *Leptospira* spp. only produced inflammatory lesions in kidneys [6]. In conclusion, the information about the pathogenesis in natural infection of rats and mice is scarce, however, the results from this study suggest that pathogenic *Leptospira* spp. are capable of producing tissue damage, but it remains unclear whether naturally infected rodents do not show clinical signs or whether animals in natural conditions are simply impaired, not trapped, or die. Nevertheless, to include histopathology and other diagnostic techniques for detecting infections should be considered as an important complementary resource for future studies.

## 5. Conclusions

A relatively low proportion of seroreactor rodents trapped in dairy farms in the Los Ríos Region, Southern Chile, but IHC and qPCR contributed to the identification of the presence of pathogenic *Leptospira* spp. in related histological lesions and 50% more infections than serology; therefore, using all tests, 64% of the rodents trapped were infected. Tubulointerstitial nephritis was the most frequently found lesion in renal tissues from naturally infected rodents. Rodents trapped during the fall had a lower risk of infection than those trapped during the rest of the year, and synanthropic species had a lower risk of infection in comparison with wildlife species.

## Figures and Tables

**Table 1:** Description of the characteristics of the different rodents trapped in dairy farms in the Los Ríos Region, Chile.

Species		Age		TOTAL	%
		A	J		
Synanthropic	*RN*	18 ^a^	23	41	33.9
	*RR*	11 ^a^	18	29	24.0
	*MM*	2 ^a^	6	8	6.6
	Subtotal	31 ^b^	47	78	64.5
Wild	*AL*	5 ^a^	2	7	5.8
	*AO*	16 ^a^	20	36	29.7
	Subtotal	21 ^b^	22	43	35.5
	TOTAL	52	69	121	
	%	43	57		100.0

RN = *Rattus norvegicus*, RR = *Rattus rattus*, MM = *Mus musculus*, AO = *Abrothrix olivaceus*, AL = *Abrothrix longipilis*. A = adults, J = juveniles, F female, M male. Equal letters indicate nonsignificant statistical differences.

**Table 2:** Main histopathological findings from 119 rodents trapped in the Los Ríos Region, Chile, whose histology was consistent with infection in any sample. Note that an animal could have more than one lesion in each organ.

Organ	Type of Disturbance	Type of Lesion	Severity	*n* *	% ^†^
Kidney	*Inflammation*	Tubulointerstitial nephritis	Mild	54	44.6
			Moderate	21	17.4
			Severe	5	4.1
	*Circulatory alterations*	Hemorrhage		42	34.7
		Congestion		2	1.7
	*Necrosis*			4	3.3
Liver	*Inflammation*	Mononuclear inflammatory infiltrate		48	39.7
	*Circulatory alterations*	Hemorrhage		15	12.4
		Congestion		11	9.1
	*Necrosis*	Necrosis		42	34.7
	*Other*	Decreased cohesion of hepatocytes		4	3.3
		Tumefaction		6	5.0
Lung	*Inflammation*	Mononuclear inflammatory infiltrate		58	47.9
	*Circulatory alterations*	Hemorrhage		80	66.1
		Congestion		42	34.7
	*Other*	Hypertrophy of arterial walls		10	8.3
Heart	*Inflammation*	Interstitial myocarditis		31	26.1
	*Circulatory alterations*	Hemorrhage		5	4.2
	*Necrosis*			1	0.8

*n* *= number of rodents that the lesion was found; % ^†^ = number of rodents that the lesion was found/number of rodents with at least 1 lesion (119).

**Table 3:** Detection of pathogenic *Leptospira* by immunohistochemistry (IHC), real-time polymerase chain reaction (qPCR), and IHC plus qPCR in rodents from different species trapped in dairy farms in the Los Ríos Region, Chile.

Variable	Category	N	IHC+		qPCR+		IHC+ and/or qPCR+	
			*n*	%	*n*	%	+	%
Species	*RN*	41	9	22.0	16	39.0	20	48.8
	*RR*	28	6	21.4	15	53.6	17	60.7
	*MM*	8	2	25.0	5	62.5	5	62.5
	*AO*	35	18	51.4	19	54.3	29	82.9
	*AL*	7	3	42.9	4	57.1	5	71.4
TOTAL		119	38	31.9	59	49.6	76	63.9

RN = *Rattus norvegicus*, RR = *Rattus rattus*, MM = *Mus musculus*, AO = *Abrothrix olivaceus*, AL = *Abrothrix longipilis*; N = total number of rodents trapped of each species.

**Table 4:** Detection of pathogenic *Leptospira* spp. by IHC, qPCR, and IHC plus qPCR together in different organs from rodents trapped in dairy farms in the Los Ríos Region, Chile.

Organ	Slides with Compatibles Lesions (H&E) (*n*)	IHC +		qPCR +		IHQ and qPCR+	
		*n*	%	*n*	%	*n*	%
Kidney	106	23	21.7	48	45.3	60	56.6
Lung	105	17	16.2	15	14.3	30	28.6
Heart	34	2	5.9	2	5.9	4	11.8
Liver	78	7	9.0	4	5.1	9	11.5
Total	323	49	15.2	69	21.4	103	31.9

**Table 5:** Frequency of infected with pathogenic *Leptospira* spp. rodents in dairy farms in the Los Ríos Region, Chile.

Variable	Category	N	MAT+		IHQ/PCR+	Infected
			*n*	*n*	*n*	%
Species	*RN*	41	2	20	21	51.2
	*RR*	29	2	17	17	58.6
	*MM*	8	0	5	5	62.5
	*AO*	36	9	29	29	80.5
	*AL*	7	1	5	5	71.4
	Total	121	14	76	77	63.6

RN = Rattus norvegicus, RR = Rattus rattus, MM = Mus musculus, AO = Abrothrix olivaceus, AL = Abrothrix longipilis; N = total number of individuals trapped.

**Table 6:** Infection status by pathogenic *Leptospira* and histopathological findings by species in rodents trapped in dairy farms in the Los Ríos Region, Chile.

Species	N	Organ/Histopathological Finding							
		Kidney (IN)		Liver (IMI)		Lungs (HAE)		Heart (IMI)	
		*Inf.*	*NI*	*Inf.*	*NI*	*Inf.*	*NI*	*Inf*	*NI*
*RN*	41	12/21	12/20	4/21	5/20	12/21	11/20	4/21	4/20
*RR*	29	11/17	4/12	7/17	3/12	10/17	7/12	8/17	4/12
*MM*	8	5/5	2/3	0/5	0/3	5/5	2/3	0/5	0/3
*AL*	36	4/5	0/2	3/5	2/2	3/5	2/2	0/5	0/2
*AO*	7	25/29	4/7	21/29	3/7	23/29	5/7	11/29	0/7
Total	121	57/77	22/44	35/77	13/44	53/77	27/44	23/77	8/44

IN = interstitial nephritis, IMI = inflammatory mononuclear infiltration, HAE = hemorrhage, Inf. = infected, NI = no infected, RN = *Rattus norvegicus*, RR = *Rattus rattus*, MM = *Mus musculus*, AO = *Abrothrix olivaceus,* AL = *Abrothrix longipilis.*

**Table 7:** Infection status by pathogenic *Leptospira* and histopathological findings by species and sex in rodents trapped in dairy farms in the Los Ríos Region, Chile.

Species	Sex	N	Organ/Histopathological Finding							
			Kidney (IN)		Liver (IMI)		Lungs (HAE)		Heart (IMI)	
			*Inf*	*N* *I*	*Inf*	*N* *I*	*Inf*	*N* *I*	*Inf*	*N* *I*
*RN*	*F*	17	3/8	5/9	2/8	1/9	4/8	4/9	2/8	1/9
	*M*	24	9/13	7/11	2/13	4/11	8/13	7/11	2/13	3/11
*RR*	*F*	11	7/9	0/2	5/9	1/2	6/9	1/2	3/9	0/2
	*M*	18	4/8	4/10	1/8	2/10	4/8	6/10	5/8	4/10
*MM*	*F*	6	4/4	1/2	0/4	0/2	4/4	2/2	0/4	0/2
	*M*	2	1/1	1/1	0/1	0/1	1/1	0/1	0/1	0/1
*AL*	*F*	1	0/0	0/1	0/0	1/1	0/0	1/1	0/0	0/1
	*M*	6	4/5	0/1	3/5	1/1	3/5	1/1	0/5	0/1
*AO*	*F*	14	10/11	1/3	6/11	0/3	7/11	2/3	6/11	0/3
	*M*	22	15/18	3/4	15/18	3/4	16/18	3/4	5/18	0/4
**Total**		121	57/77	22/44	35/77	13/44	53/77	27/44	23/77	8/44

IN = interstitial nephritis, IMI = inflammatory mononuclear infiltration, HAE = hemorrhage, Inf. = infected, NI = no infected, RN *= Rattus norvegicus*, RR = *Rattus rattus*, MM = *Mus musculus,* AO = *Abrothrix olivaceus*, AL = *Abrothrix longipilis,* F = female, M = male.

**Table 8:** Infection status by pathogenic *Leptospira* and histopathological findings by species and age in rodents trapped in dairy farms in the Los Ríos Region, Chile.

Species	Age	N	Organ/Histopathological Finding							
			Kidney (IN)		Liver (IMI)		Lungs (HAE)		Heart (IMI)	
			*Inf*	*NI*	*Inf*	*NI*	*Inf*	*NI*	*Inf*	*NI*
*RN*	*A*	18	4/10	5/8	1/10	2/8	9/10	5/8	1/10	2/8
	*J*	23	8/11	7/12	3/11	3/12	3/11	6/12	3/11	2/12
*RR*	*A*	11	5/7	2/4	3/7	2/4	5/7	4/4	2/7	0/4
	*J*	18	6/10	2/8	4/10	1/8	5/10	3/8	6/10	4/8
*MM*	*A*	2	1/1	0/1	0/1	0/1	1/1	1/1	0/1	0/1
	*J*	6	4/4	2/2	0/4	0/2	4/4	1/2	0/4	0/2
*AL*	*A*	5	4/5	0/0	3/5	0/0	3/5	0/0	0/5	0/0
	*J*	2	0/0	0/2	0/0	2/2	0/0	2/2	0/0	0/2
*AO*	*A*	16	13/14	1/2	8/14	1/2	11/14	1/2	6/14	0/2
	*J*	20	12/15	3/5	13/15	2/5	12/15	4/5	5/15	0/5
**Total**		121	57/77	22/44	35/77	13/44	53/77	27/44	23/77	8/44

IN = interstitial nephritis, IMI = inflammatory mononuclear infiltration, HAE = hemorrhage, Inf. = infected, NI = no infected, RN *= Rattus norvegicus*, RR = *Rattus rattus*, MM = *Mus musculus,* AO = *Abrothrix olivaceus*, AL = *Abrothrix longipilis*, A = adults, J = juveniles.

**Table 9:** Conditional mixed-logistic regression model results showing risk factors for rodents infected by pathogenic *Leptospira* spp. and trapped in dairy farms in the Los Ríos Region, Chile.

Variable	Category	OR	95% CI	*p*-Value
Season	*Winter*	Ref.		
	*Fall*	0.29	0.08; 0.94	0.04
	*Spring*	0.44	0.13; 1.34	0.16
	*Summer*	0.98	0.28; 3.76	0.98
Species	*Wildlife*	Ref.		
	*Synanthropic*	0.22	0.07; 0.65	<0.001

Akaike Information Criteria (AIC) = 154.5.

## Supplementary Materials

The following are available online at https://www.mdpi.com/2076-2615/10/11/2133/s1, Table S1: Histological findings related to infection status in rodents trapped in dairy farms in the Los Ríos Region, Chile.
