# Supplementary material for: Assessment of Risk Factors in Synanthropic and Wild Rodents Infected by Pathogenic Leptospira spp. Captured in Southern Chile
Source: Animals (Basel). 2020 Nov 17;10(11):2133. doi: 10.3390/ani10112133 (PMC7697743; doi:10.3390/ani10112133)
Supplement: Supplementary file 1 [file animals-10-02133-s001.pdf]

animals

MDPI

Table S1. Histological findings related to infection status in rodents trapped in dairy farms at Los Ríos region, Chile.

Organ	Lesion	Infected (%)	No infected (%)
Kidney	Interstitial nephritis	74.0	50.0
	Hemorrhage	36.4	31.8
Lung	Hemorrhage	68.8	61.4
	Congestion	31.2	40.9
	Inflammatory mononuclear infiltration	49.4	45.5
Liver	Inflammatory mononuclear infiltration	45.5	29.5
	Hemorrhage	9.1	18.2
	Congestion	9.1	9.1
	Necrosis	28.6	45.5
Heart	Inflammatory mononuclear infiltration	29.9	18.2

Publisher's Note: MDPI stays neutral with regard to jurisdictional claims in published maps and institutional affiliations.

© 2020 by the authors. Licensee MDPI, Basel, Switzerland. This article is an open access article distributed under the terms and conditions of the Creative Commons Attribution (CC BY) license (http://creativecommons.org/licenses/by/4.0/).
